# Taurine as Antioxidant in a Novel Cell- and Oxygen Carrier-Free Perfusate for Normothermic Machine Perfusion of Porcine Kidneys

**DOI:** 10.3390/antiox12030768

**Published:** 2023-03-21

**Authors:** Laura Zarnitz, Benedict M. Doorschodt, Lisa Ernst, Aisa Hosseinnejad, Eileen Edgworth, Tamara Fechter, Alexander Theißen, Sonja Djudjaj, Peter Boor, Rolf Rossaint, René H. Tolba, Christian Bleilevens

**Affiliations:** 1 Department of Anesthesiology, Medical Faculty, University Hospital RWTH Aachen, 52074 Aachen, Germany; 2 Institute for Laboratory Animal Science and Experimental Surgery, Faculty of Medicine, RWTH Aachen University, 52074 Aachen, Germany; 3 DWI—Leibniz-Institute for Interactive Materials e.V., 52056 Aachen, Germany; 4 Institute of Pathology & Division of Nephrology, Medical Faculty, RWTH Aachen, 52074 Aachen, Germany

**Keywords:** normothermic machine perfusion, kidney preservation, ischemia reperfusion injury, oxygen carrier-free perfusion, cell free perfusion, taurine

## Abstract

Donor organ-shortage has resulted in the increased use of marginal grafts; however, normothermic machine perfusion (NMP) holds the potential for organ viability assessment and restoration of marginal grafts prior to transplantation. Additionally, cell-, oxygen carrier-free and antioxidants-supplemented solutions could potentially prevent adverse effects (transfusion reactions, inflammation, hemolysis), associated with the use of autologous packed red blood cell (pRBC)-based perfusates. This study compared 6 h NMP of porcine kidneys, using an established pRBC-based perfusate (pRBC, n = 7), with the novel cell- and oxygen carrier-free organ preservation solution Ecosol, containing taurine (Ecosol, n = 7). Despite the enhanced tissue edema and tubular injury in the Ecosol group, related to a suboptimal molecular mass of polyethylene glycol as colloid present in the solution, functional parameters (renal blood flow, intrarenal resistance, urinary flow, pH) and oxygenation (arterial pO_2_, absence of hypoxia-inducible factor 1-alpha) were similar to the pRBC group. Furthermore, taurine significantly improved the antioxidant capacity in the Ecosol group, reflected in decreased lactate dehydrogenase, urine protein and tubular vacuolization compared to pRBC. This study demonstrates the feasibility of 6 h NMP using a taurine containing, cell- and oxygen carrier-free perfusate, achieving a comparable organ quality to pRBC perfused porcine kidneys.

## 1. Introduction

The only curative treatment modality for end stage renal disease, reducing all-cause mortality, remains kidney transplantation [1,2]. According to Eurotransplant data, 4002 kidneys were transplanted in 2021, whilst 10,269 patients were enrolled on active waiting lists to receive a kidney graft at the end of 2021 and 706 people died awaiting kidney transplant [3]. To increase the pool of kidneys suitable for transplantation, expanded criteria for donation after brain death (ECD-DBD) as well as donation after circulatory death donors (DCD) are more often being employed. However, the organs from these donor categories are associated with higher incidences of primary non-function (PNF), delayed graft function (DGF) and graft loss [4,5,6], and thus defined as marginal grafts.

Over the last decade, normothermic machine perfusion (NMP) is increasingly investigated in preclinical as well as clinical studies since it holds a true potential of increasing the viability of marginal grafts. NMP also offers the possibility of viability assessment and pharmaceutical intervention prior to transplantation, in contrast to static cold storage (SCS) or hypothermic machine perfusion (HMP) [7,8]. As shown previously in cohort studies, NMP is capable of reducing PNF and DGF and improving immediate kidney function [9,10,11,12,13].

The adequate supplying of the kidney with nutrients and oxygen is vital to maintain the metabolism and organ function during NMP. The optimal composition of perfusion solutions for NMP remains to be defined. Although a standard NMP protocol is lacking, perfusates derived from whole blood or packed red blood cells (pRBC) combined with a buffer solution are most commonly applied [14,15]. As perfusion with whole blood or pRBC’s bears the risk of transfusion reactions through increased immunogenicity, inflammation, and hemolysis due to perfusion using systems with artificial surfaces, alternatives are the focus of experimental studies. In an attempt to replace pRBCs in our established NMP perfusate, we recently described the use of Hemoglobin-Based Oxygen Carrier-301 (HBOC-301, HBO2 Therapeutics, Souderton, PA, USA) [16] showing HBOC-301 to be inferior to pRBC’s in a 6 h porcine kidney NMP setting. Cell free solutions, such as Lifor, Aqix RS-I, University of Wisconsin solution (UW) and Steen solution have recently been described as alternative NMP perfusates in experimental studies [17,18,19,20]. It was shown that neither cells nor artificial oxygen carriers are required for adequate tissue oxygenation during 2 h NMP [17,20].

In the current study, we aimed to investigate the use of Ecosol organ preservation solution (TX Innovations B.V., Gulpen, The Netherlands), originally developed for kidney SCS, as an alternative to our previously established pRBC-based NMP perfusate [16,21,22]. In contrast to HBOC-301, Ecosol is an acellular and oxygen carrier-free solution. For DBD and DCD kidney SCS with subsequent normothermic reperfusion as well as for venous systemic oxygen persufflation and HMP, Ecosol showed an improved preservation quality compared to Histidine-Tryptophan-Ketoglutarate solution (HTK) [23,24].

As inflammatory mediators are upregulated during NMP, reactive oxygen species (ROS) are formed, resulting in tissue damage [25]. Ecosol contains taurine, a potent antioxidant, to minimize oxidative damage. Taurine supplementation to perfusion solutions previously demonstrated effectiveness in oxygenated liver preservation, reducing lipid peroxidation and vascular resistance [26,27]. It was also shown to preserve the renal function [28] and ameliorate liver injury in a rat cholestasis model [29].

This study aims to show the beneficial effects of a cell- and oxygen carrier-free, taurine-containing perfusate compared to the widely used pRBC-based perfusate in a porcine model of DCD kidney NMP.

## 2. Materials and Methods

### 2.1. Experimental Protocols

The experimental protocol was approved by the Institutional Animal Care and Use Committee of the RWTH Aachen University Hospital and performed in accordance with German legislation governing animal studies following the “Guide for the care and use of Laboratory Animals” (NIH publication, 8th edition, 2011) and the Directive 2010/63/EU on the protection of animals used for scientific purposes (Official Journal of the European Union, 2010).

Kidneys were retrieved and either perfused with Ringer’s Solution for Infusion (B. Braun Melsungen AG, Melsungen, Germany) based perfusion solution mixed with autologous pRBCs or with Ecosol. The two experimental groups were defined as perfusion with one of the perfusates, and henceforward will be referred to as pRBC and Ecosol. Kidney pairs were perfused simultaneously for 6 h with perfusate and urine sample collection at regular time points. Biopsies and tissue samples were taken before and after perfusion for histology and molecular biology analysis.

### 2.2. Preparation of Kidney Grafts

Seven German landrace pigs, 56.3 ± 2.7 kg (MW ± SEM) body weight, from a disease-free barrier breeding facility, were housed in fully air-conditioned rooms (22 °C room temperature, 50% relative humidity). For a minimum of seven days, they were allowed to acclimatize to these conditions and fasted for 12 h prior to surgery with free access to water. The animals were premedicated with 15 mg/kg BW ketamine (Ceva GmbH, Duesseldorf, Germany), 8 mg/kg BW azaperone (Stresnil^®^, Janssen-Cilag GmbH, Neuss, Germany), and 10 mg atropine (1 mL/1% atropine sulfate, Dr. Franz Köhler Chemie GmbH, Bensheim, Germany) administrated intramuscularly. Euthanization was performed using 1 mL/kg BW pentobarbital (Narcoren, Merial GmbH, Hallbergmoss, Germany), cardiac arrest was testified, and a midline laparotomy was performed. Immediately after cardiac arrest, the vena cava was cannulated and 450 mL of venous blood were collected into sterile blood bags (Composelect^®^, Fresenius Kabi Austria GmbH, Graz, Austria) each and prepared for pRBC production as described previously by our working group [16]. Kidneys were explanted simultaneously and weighed. For washout, 250 mL of warm (37 °C) Ringer’s solution or Ecosol were supplemented with 5000 IU heparin (B. Braun Melsungen AG, Melsungen, Germany). Directly after explantation, 100 mL of the flushing solution were administered, the other 150 mL after cannulation of the renal artery (Retrograde Cardioplegia Catheter, 14 Fr., Edwards Life Sciences, RC014; Unterschleißheim, Germany). Cannulation of the renal vein (Thomafluid^®^ Luer Lock Tubing Adapter Female together with a flexible tube, ID 6.4 mm (1/4′′), Reichelt Chemietechnik GmbH + Co., Heidelberg, Germany) and ureter (Suction Catheter ProFlo straight tip with a funnel, 14 Ch., ConvaTec GmbH, Munich, Germany) were performed to enable sample collection. In accordance with the 3R principle in experimental animal sciences (Refine, Reduce, Replace), all remaining organs and tissues of the experimental animals were used by other in-house institutes and groups.

### 2.3. Normothermic Machine Perfusion

Kidneys were perfused simultaneously for 6 h at a mean arterial pressure (MAP) of 75 mmHg and at a temperature of 36.5 ± 0.1 °C, according to our established protocol [16,21,22].

**Figure 1:**
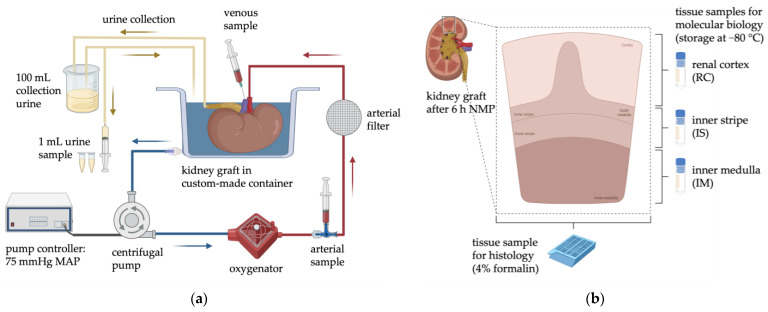
(**a**) Schematic arrangement of the NMP circuit and the sample collection during perfusion. (**b**) Collection of tissue samples for both histology and biomolecular analysis. Created with BioRender.com (accessed on 24 January 2023).

The perfusion circuits (Figure 1a) consisted of a custom-made container for the kidneys, wherein the kidney was emersed in the effluent instead of a humid chamber, a centrifugal blood pump (Affinity CP, Medtronic GmbH, Meerbusch, Germany), an oxygenator (Newborn A.L.ONE ECMO, Eurosets GmbH, Gröbenzell, Germany), and a pediatric arterial filter (Affinity^TM^, Medtronic GmbH, Meerbusch, Germany).

Kidneys were connected to the circuit via the renal artery, with the effluent draining freely from the renal vein into the container, which served as a reservoir for the perfusion loop. The centrifugal blood pump was driven by a custom-designed pump controller (Informatik 11-Embedded Software, RWTH Aachen University, Aachen, Germany) automatically adjusting the rotations per minute (RPM) according to a preset arterial blood pressure. Within the first five minutes, starting at 25 mmHg, the MAP was continuously increased to the target MAP of 75 mmHg and held throughout the 6 h NMP. The perfusate was oxygenated through a heated oxygenator and ventilated with 0.5 L/min carbogen (95% O_2_/5% CO_2_). The temperature was maintained at 36.5 °C by a water bath thermostat connected to the oxygenator. The MAP and temperature were monitored (IntelliVue MX500, Royal Philips Electronics, Amsterdam, The Netherlands) continuously, and the arterial renal blood flow (RBF) was recorded through an ultrasonic flow meter (SonoTT, em-tec GmbH, Finning, Germany).

According to Weißenbacher et al., the urine was recirculated for improved metabolic homeostasis and to keep the perfusate volume within the circuit constant [30].

### 2.4. Perfusion Solutions

In both experimental groups, a total perfusate volume of 1000 mL was provided by using either 500 mL autologous pRBC and 500 mL of a Ringer’s solution-based buffer as previously described by our working group [16], or 1000 mL Ecosol. In the Ecosol group, 0.058 g of dissolved creatinine (Sigma Aldrich Chemie GmbH, Taufkirchen, Germany) was added, matching the concentration in the pRBC group to enable creatinine clearance (CrCl) determination.

Both groups were supplemented with 750 mg cefuroxime (Dr. Friedrich Eberth Arzneimittel GmbH, Ursensollen, Germany), 5000 IU heparin, 8 mg dexamethasone (Fortecortin inject, Merck KgaA, Darmstadt, Germany), and sodium bicarbonate 8.4% (Deltamedica GmbH, Reutlingen, Germany) titrated until a pH of 7.318 ± 0.009 was reached (12.7 ± 0.6 mL).

Syringe pumps continuously administered a nutritive solution at a rate of 22.4 mL/h to pRBC, consisting of 44 mL Nutriflex (B. Braun Melsungen AG, Melsungen, Germany), 0.4 mL Cernevit (Baxter Deutschland GmbH, Unterschleißheim, Germany) and 20 IU insulin glulisine (Apidra, Sanofi-Aventis Deutschland GmbH, Frankfurt am Main, Germany). Since Ecosol contains a unique mixture of additives and nutrients [24], the nutritive solution added to the pRBC group was not added to the Ecosol group. Instead, 44.4 mL Ecosol supplemented with 20 IU insulin was infused at the same rate, to match the volumes added to the pRBC group.

### 2.5. Biochemistry

As a baseline sample, 1 mL of the initial perfusate was taken before the kidneys were attached to the primed circuit (0 min). Arterial, venous and urine samples were collected at 8 distinct time points (5, 30, 60, 120, 180, 240, 300, and 360 min). The urine output was collected between these time points, and as soon as 100 mL was produced, 1% of the volume was collected. The remaining amount was recirculated into the reservoir container.

Blood gas analysis was performed by using an in-line blood gas analyzer (ABL 800Flex, Radiometer GmbH, Krefeld, Germany). Arterial, venous and urine samples were analyzed for pH, partial pressure of oxygen (pO_2_), partial pressure of carbon dioxide (pCO_2_), saturation of oxygen (sO_2_), concentration of total hemoglobin (ctHb), electrolytes, glucose, and lactate.

Arterial samples were centrifuged at 10,000 rpm, 4 °C, for 10 min. Aliquots of the supernatant were stored at −80 °C for biochemistry analysis of aspartate aminotransferase (AST), lactate dehydrogenase (LDH), total protein, urea, creatinine, and iron. Urine samples were stored alike and analyzed for creatinine, urea, and total protein concentrations. Biochemistry analysis was performed by the local ISO 9001:2015-certified laboratory at the Institute of Laboratory Animal Science and Experimental Surgery.

Kidney weights were recorded before and after perfusion. Temperature, pump rpm, MAP, RBF, and total urine output were documented for each time point. Intrarenal resistance (IRR) was calculated as MAP/RBF/100 g and CrCl as urine creatinine × urinary flow/plasma creatinine/100 g.

### 2.6. Molecular Biomarkers

After perfusion, tissue samples of the renal cortex (RC), inner stripe (IS) and inner medulla (IM) were snap-frozen and stored at −80 °C for molecular biology (Figure 1b).

Serum and tissue concentrations of Hypoxia Inducible Factor-1alpha (HIF-1α, Porcine HIF-1α ELISA Kit; MBS263046, San Diego, CA, USA), and serum levels of Interleukin-6 (IL-6, Porcine IL-6 Quantikine ELISA Kit, P6000B, R & D Systems, Inc., Minneapolis, MN, USA) were analyzed through enzyme-linked immunosorbent assays (ELISA) by preparing 40 mg of tissue according to the manufacturer’s instructions. All samples were diluted 1:2 and plates were read at 450 nm with the iMark™ Microplate Absorbance Reader (Bio-Rad Laboratories GmbH, Feldkirchen, Germany).

For Western Blot (WB) analysis, Radioimmunoprecipitation Assay buffer (RIPA, Lysis Buffer, 10×, Merck KGaA, Darmstadt, Germany) was supplemented with 1% Sodium dodecyl sulfate solution (BioUltra for molecular biology, Sigma-Aldrich Chemie GmbH, Taufkirchen, Germany), PhosSTOP (Roche GmbH, Grenzach-Wyhlen, Germany) and complete Mini (Roche GmbH, Grenzach-Wyhlen, Germany). Then, 40 mg of tissue was lysed with 800 µL of the prepared RIPA buffer. After homogenization on ice, samples were loaded onto QIAshredder columns (QIAGEN GmbH, Hilden, Germany) and centrifuged at 2000 rpm at 4 °C for 2 min. Lysates were stored at −80 °C until analysis.

Next, 5 µL lysate was mixed with 5.5 µL Laemmli buffer (4x Laemmli Sample Buffer, #1610747, Bio-Rad Laboratories GmbH, Feldkirchen, Germany), 2.2 µL dithiothreitol and 9.3 µL aqua destillata, respectively. Prepared samples were boiled at 100 °C for 5 min. Then, 15 comb 12% gels (TGX Stain-Free™ FastCast™ Acrylamide Kit, 12%, #1610185, Bio-Rad Laboratories GmbH, Feldkirchen, Germany) were loaded with 20 µL of the sample per lane. Proteins were then separated through sodium dodecyl sulfate polyacrylamide gel electrophoresis (SDS-PAGE). Using the ChemiDoc™ MP Imaging System (Bio-Rad Laboratories GmbH, Feldkirchen, Germany), stain-free shots were visualized. With the Trans-Blot^®^ Turbo™ Transfer System (Bio-Rad Laboratories GmbH, Feldkirchen, Germany), proteins were transferred onto Polyvinylidenfluorid (PVDF) membranes and then blocked with 5% bovine serum albumin for 1 h at room temperature. Membranes were incubated with primary antibodies (Caspase-3: #14220; phosphorylated Erk1/2: #9101; Erk1/2: #9102; pAkt: #9271; Akt: #4691; Cell Signaling Technology, Leiden, The Netherlands; Vinculin: V9131, Sigma-Aldrich Chemie GmbH, Taufkirchen, Germany) for 2 h at room temperature or overnight at 4 °C, followed by appropriate secondary antibodies (anti-rabbit: #7074, Cell Signaling Technology, Leiden, The Netherlands; anti-mouse: GENXA931-1ML, Sigma-Aldrich Chemie GmbH, Taufkirchen, Germany). Bands were visualized by use of ECL (ECL™ Prime Western-Blot-System, GERPN22332, Sigma-Aldrich Chemie GmbH, Taufkirchen, Germany). Integrated density values (IDV) for each protein band were calculated using the Image Lab software (Bio-Rad Laboratories GmbH, Feldkirchen, Germany), and normalized to the total protein amount using Stainfree technology, as described previously [31].

### 2.7. Oxidative Stress

Serum samples were tested for oxidative stress by measuring the static oxidation-reduction potential (ORP) and the antioxidant capacity (AC) using the RedoxSYS Diagnostic SystemTM (Aytu BioScience, Inc., Englewood, CO, USA). This method was previously established by our group [16,22].

### 2.8. Histology

Needle biopsies were taken using a 14G Tru-Cut needle (Merit Medical Systems, South Jordan, UT, USA) before perfusion, fixed in 4% buffered formalin for 7 days, and embedded in paraffin. Tissue samples from renal cortex and medulla, collected after 6 h NMP (Figure 1b), were processed similarly. Four-micron sections were stained with periodic acid–Schiff reaction (PAS).

All analyses were performed by a senior pathologist, blinded for timepoint and group. The tubular injury was assessed on a scale of 0–3 as follows: (0) no injury; (1) slight tubular injury; (2) prominent tubular injury; (3) and necrotic cell injury. Tubular vacuolization was determined dependent on the percentage of vacuolization-affected tubules on a scale of 0–4; (0) 0–1%; (1) 2–15%; (2) 16–50%; (3) 51–75%; and (4) 76–100%.

### 2.9. Characterization of Ecosol before and after Perfusion

During perfusion, gel formation occurred on kidneys perfused with Ecosol, therefore, the composition of Ecosol and the structure of the formed gel after 6 h NMP using Ecosol were characterized by Fourier Transform Infrared (FTIR) spectroscopy with Thermo Nicolet Nexus 470 FTIR spectrometer (Thermo Fisher Scientific Inc., Waltham, MA, USA) on Attenuation Total Reflection (ATR) mode using silicon crystal. Data were analyzed using the Origin Pro (version 9.5.5) software.

Gel Permeation Chromatography (GPC) measurements were further used to analyze the molecular weights of the aqueous Ecosol components, which were taken on one precolumn (8 × 50 mm) and three Suprema-Lux gel columns (8 × 300 mm) at 40 °C at a flow rate of 1.0 mL min^−1^ using an Agilent 1200 system. The diameter of the gel particles measured 5 μm, where the nominal pore widths were 30, 1000, and 1000 Å. An aqueous solution of 0.05 wt% sodium azide (NaN_3_) was used as an eluent. Calibration was achieved using narrow-distributed polyethylene glycol (PEG) standards. The GPC data were analyzed using the WinGPC UniChrom program (version 8.3.2).

Prior to analysis, Ecosol was purified by dialysis against distilled water for a day and freeze-dried. Likewise, the formed gel mass was thoroughly washed with distilled water followed by freeze-drying before the measurement.

### 2.10. Statistical Analysis

Statistical analysis was performed using GraphPad Prism 9.4.1 software package (GraphPad Software Inc., San Diego, CA, USA). After Kolmogorov–Smirnov normality test, two-way analysis of variance (ANOVA) for multiple comparisons was used followed by Šídák post-test correction for all measurements of perfusion, oxygenation, kidney injury, and oxidative stress markers. For kidney weights, WB and ELISA data, one-way ANOVA-test was carried out. Data are presented as mean ± standard error of the mean (SEM) and *p*-values < 0.05 were considered significant.

## 3. Results

### 3.1. Perfusion Parameters

**Figure 2:**
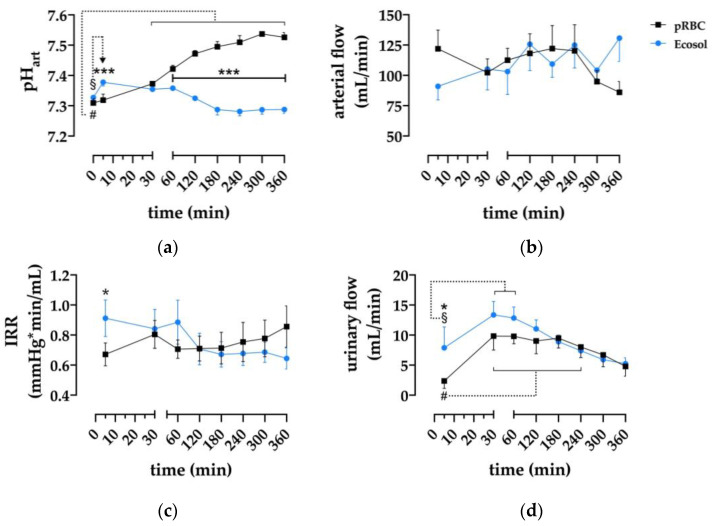
Assessment of renal perfusion parameters. (**a**) Effect of time and perfusion medium on arterial pH; (**b**) arterial flow of kidneys during perfusion; (**c**) dtatistical analysis of intrarenal resistance during machine perfusion; and (**d**) urinary flow during 6 h NMP; * *p* < 0.05, *** *p* < 0.001 (pRBC vs. Ecosol); # *p* < 0.05 (pRBC time point vs. pRBC baseline); § *p* < 0.05 (Ecosol time point vs. Ecosol baseline).

The pH was similar in both perfusates prior to connection of the kidney (pRBC; 7.309 ± 0.014 vs. Ecosol; 7.327 ± 0.012) (Figure 2a), after which the pH in the pRBC group increased significantly over time, reaching 7.526 ± 0.016 at t = 360 min and remaining significantly higher than the Ecosol group from 60 min (*p* < 0.0002). In contrast, the pH in the Ecosol group remained stable during 6 h perfusion, except at 5 min after the start of perfusion.

Arterial flow (Figure 2b) remained stable in both groups throughout 6 h NMP and did not differ at any timepoint.

Intrarenal resistance in both groups did not differ, except at t = 5 min, where Ecosol was higher compared to the pRBC group (*p* = 0.0275).

Urinary flow (Figure 2d) differed between the groups only at the start of perfusion (t = 5 min) with 2.4 ± 1.2 mL/min vs. 7.9 ± 3.5 mL/min (pRBC vs. Ecosol resp., *p* = 0.01). Peak urinary flow was reached in both groups at 30 min (9.8 ± 2.3 mL/min in pRBC and 13.4 ± 2.2 mL/min in Ecosol).

### 3.2. Oxygenation

**Figure 3:**
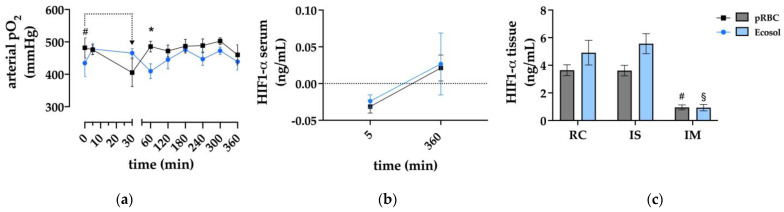
Oxygenation of kidney grafts during NMP. (**a**) Arterial pO_2_ levels; * *p* < 0.05 (pRBC vs. Ecosol); # *p* < 0.05 (pRBC 30 min vs. pRBC 0 min); (**b**) ELISA analysis of HIF1-α serum concentrations at 5 and 360 min of perfusion; and (**c**) ELISA analysis of HIF1-α tissue concentrations after 6 h NMP; # *p* < 0.05 (pRBC IM vs. pRBC RC & pRBC IS); § *p* < 0.05 (Ecosol IM vs. Ecosol RC & Ecosol IS); *RC* renal cortex; *IS* inner stripe; *IM* inner cortex.

During NMP, supraphysiological pO_2_ values were achieved in both groups with average values of 473.1 ± 9.4 mmHg in pRBC and 451.9 ± 7.6 mmHg in Ecosol (Figure 3a).

HIF-1α was used as an indicator for hypoxic conditions, as it is an important regulator for downstream processes in cells suffering from hypoxia. No significant correlation could be found between HIF-1α levels and the perfusion groups (Figure 3b,c). In RC and IS samples, HIF-1α levels were comparable, but significantly higher than those in IM tissue samples (*p* < 0.05).

### 3.3. Markers of Kidney Injury

**Figure 4:**
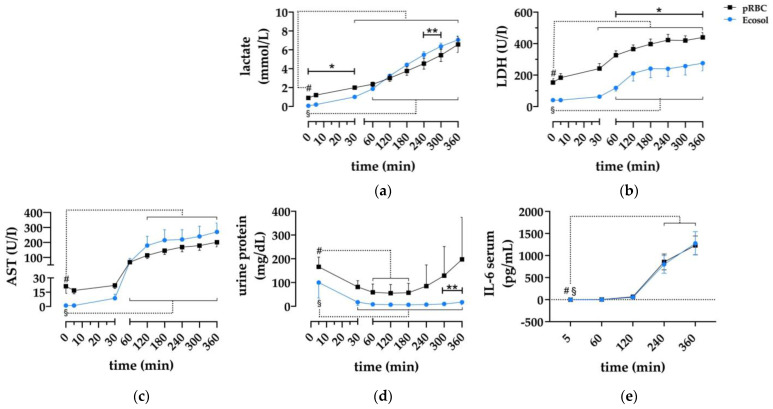
Evaluation of kidney damage parameters. (**a**–**c**) Serum concentrations of AST, LDH and lactate during 6 h NMP; (**d**) protein concentrations in urine samples of both perfusion groups; and (**e**) ELISA analysis of IL-6 concentrations in serum samples; * *p* < 0.05 (pRBC vs. Ecosol); ** *p* < 0.01 (pRBC vs. Ecosol); # *p* < 0.05 (pRBC time point vs. pRBC baseline); § *p* < 0.05 (Ecosol time point vs. Ecosol baseline).

For a rough estimation of kidney damage, perfusate concentrations of lactate, AST, LDH, and urine protein were evaluated. Lactate levels (Figure 4c) increased significantly, from 0.9 ± 0.07 to 6.57 ± 0.85 mmol/L (pRBC) and from 0.07 ± 0.02 to 7.06 ± 0.42 mmol/L (Ecosol). Within the initial 30 min of perfusion, lactate levels were significantly higher in 

pRBC, and in Ecosol from 240 to 300 min. Similarly, AST (Figure 4a) and LDH (Figure 4b) levels rose in both groups over time. There were no differences in AST levels; however, LDH levels were higher in pRBC from 60 min until the end of perfusion (*p* < 0.04). Urine protein concentrations (Figure 4d) were higher in pRBC compared to Ecosol at t = 5 (*p* < 0.01), t = 300 and t = 360 (*p* < 0.01). IL-6 levels did not differ between the groups (Figure 4e).

### 3.4. Antioxidative Properties of the Perfusates

**Figure 5:**
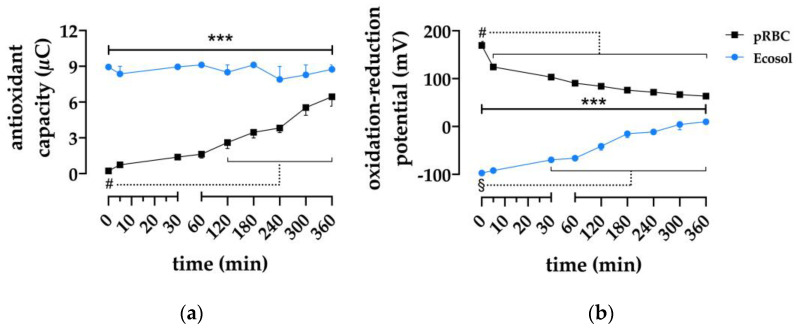
Effect of perfusate composition on antioxidative features. (**a**) Statistical analysis of antioxidant capacity and (**b**) oxidation-reduction potential; *** *p* < 0.001 (RBC vs. Ecosol); # *p* < 0.05 (RBC time point vs. RBC baseline); § *p* < 0.05 (Ecosol time point vs. Ecosol baseline).

The antioxidant capacity (Figure 5a) was higher in the Ecosol group than in the RBC group (*p* < 0.001). It remained stable until the end of perfusion in the Ecosol group, whereas it increased over time in the RBC group. The oxidation-reduction potential (Figure 5b) was lower in Ecosol perfused kidneys in contrast to pRBC during perfusion (*p* < 0.001); however, it constantly increased, as opposed to a decrease seen in the RBC group.

### 3.5. Electrolytes and Glucose

Electrolyte and glucose levels during 6 h perfusion, compared to standard values are displayed in Table 1. In both groups, K^+^ levels during 6 h perfusion were higher than the standard value (Table 1), which can partly be explained by the protocol for sacrificing experimental animals using sodium pentobarbital and potassium (782 mg) mixture. Overall, no physiological ranges of electrolytes or glucose levels were sustained for the duration of perfusion.

### 3.6. Macroscopic Kidney Appearance and Perfusate Composition

**Figure 6:**
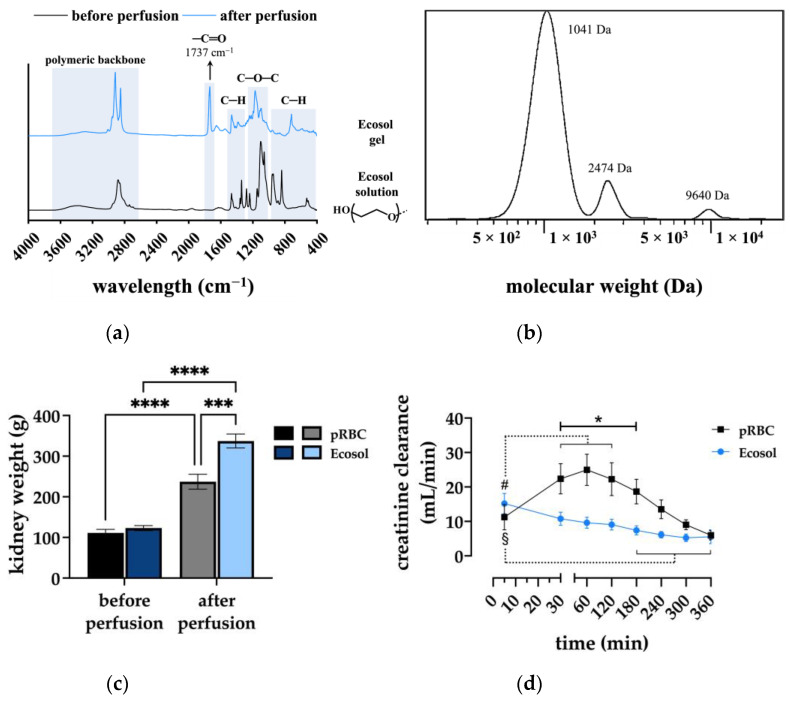
(**a**) FTIR spectra of the Ecosol solution before and after perfusion; (**b**) GPC analysis of the Ecosol solution demonstrating the M_w_ of PEG; (**c**) kidney weights of both experimental groups before and after 6 h NMP; *** *p* < 0.001; **** *p* < 0.0001; and (**d**) creatinine clearances of both groups during 6 h NMP; * *p* < 0.05 (pRBC vs. Ecosol); # *p* < 0.05 (pRBC time point vs. pRBC baseline); § *p* < 0.05 (Ecosol time point vs. Ecosol baseline).

Kidney function was approximated by creatinine clearance rates (Figure 6d). In the pRBC group, CrCl was shown to peak at 60 min with 24.98 ± 4.53 mL/min followed by a constant decrease and was higher than in Ecosol in the first 3 h of perfusion (*p* < 0.03). However, perfusate as well as urinary creatinine concentrations were lower in Ecosol perfused kidneys, suggesting loss of creatinine, as added concentrations were similar.

Additionally, formation of a gel-like mass appeared around the hilus in Ecosol perfused kidneys. After removing the mass from the kidney samples, it still maintained its gel-like properties and was insoluble in water or any other standard organic solvents at room temperature, implying an irreversible fully cross-linked structure of the mass. Since the colloidal component of Ecosol preventing extravasation of the solution is polyethylene glycol (PEG), the formation of the gel mass was hypothesized to result from the accumulation of a lower M_w_ PEG than the specified PEG 35 kDa in the Ecosol solution supplied by the manufacturer.

As shown in Figure 6a, the structure analysis of the Ecosol prior to perfusion measured by FTIR spectroscopy mainly revealed the characteristic signals of a typical non-functionalized PEG as a base component; however, the FTIR analysis of the Ecosol after perfusion (the formed gel) shows some structural changes. Particularly the presence of an additional carbonyl bond (−C=O) at 1737 cm^−1^ is evident. Furthermore, the evaluation of the Ecosol molecular weight analyzed by GPC confirmed the presence of different molecular weights with a M_w_ of 1–10 kDa and the absence of higher M_w_ PEG (Figure 6b).

The inhomogeneous composition of the Ecosol due to the wrong-sized polymers or any other polymer impurities might explain the unexpected behavior of PEG leading to crosslinking (gelation). Possibly, creatinine was further intercepted by the formed Ecosol gel as the presence of carbonyl in the gel structure can be correlated to the interaction with creatinine. Therefore, it led to the lack of efficient creatinine clearance in the Ecosol group.

Kidney weights (Figure 6c) did not differ in both experimental groups prior to perfusion. However, after perfusion kidney weights increased significantly compared to the initial weights (*p* < 0.0001). Moreover, kidney weights after perfusion differed significantly between the groups, with a significantly higher weight gain in the Ecosol group (*p* < 0.001).

### 3.7. Apoptosis in Different Areas of the Kidney after NMP

**Figure 7:**
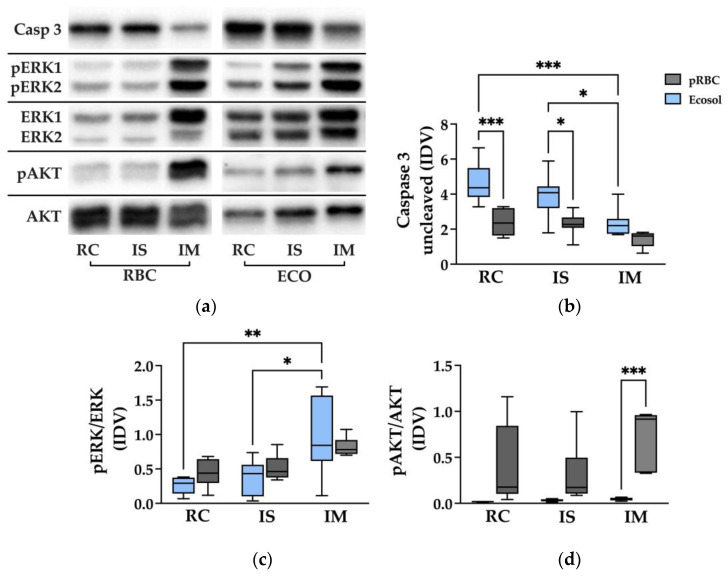
Western Blot analysis of Casp 3, pERK/ERK and pAkt/Akt. (**a**) Representative WB analysis of different kidney areas in both groups; and (**b**–**d**) statistical analysis of Casp 3, pERK/ERK and pAkt/Akt protein levels; * *p* < 0.05, ** *p* < 0.01; *** p < 0.001; *VIN* Vinculin; *Casp 3* Caspase 3; *ERK1/2* extracellular-signal regulated kinases 1/2; *pERK1/2* phosphorylated extracellular-signal regulated kinases 1/2; *Akt* protein kinase B; *pAkt* phosphorylated protein kinase B; *IDV* integrated density value; *RC* cortex; *IS* inner stripe; *IM* inner medulla.

For the determination of apoptosis, uncleaved Casp 3, phosphorylated ERK to ERK (pERK/ERK) and phosphorylated AKT to AKT (pAKT/AKT) ratios were measured (Figure 7a–d).

Overall, the levels of uncleaved Casp 3 were higher in the Ecosol group than in the pRBC group. Levels of Casp 3 in RC and IS samples were significantly lower in pRBC compared to Ecosol kidneys (*p* < 0.03). Significantly less Casp 3 was found in IM than in RC and IS samples of the Ecosol group (*p <* 0.05). Cleaved Casp 3 could not be found in any sample.

The levels of pERK/ERK showed no difference between the Ecosol and pRBC groups. Within Ecosol, pERK/ERK was significantly higher in IM compared to RC as well as IS tissue samples (*p* < 0.01).

In samples of IM, pAKT/AKT was significantly higher in pRBC kidneys than in Ecosol kidneys (*p* = 0.0005). RC and IS samples showed a tendency for pAKT/AKT being higher in pRBC, which could not be confirmed by statistical significance.

### 3.8. Tissue Damage as Observed through Pathology

**Figure 8:**
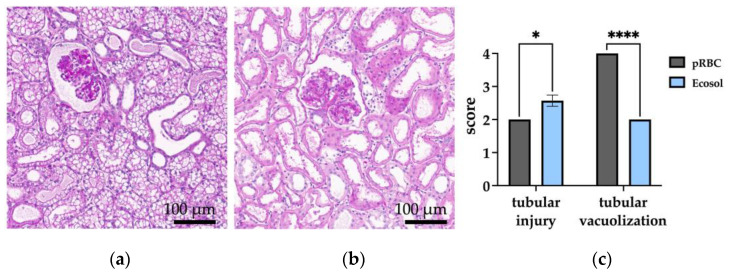
Histological assessment of kidneys after perfusion. (**a**) Exemplary PAS-stained cross-sections of pRBC and (**b**) Ecosol (40-fold magnified); and (**c**) statistical analysis of tubular injury and tubular vacuolization scores after perfusion; * *p* < 0.05; **** *p* < 0.0001.

All kidneys showed diffuse and prominent acute tubular damage with different extents of tubular vacuolization (Figure 8a,b). The most common finding was tubular dilatation, loss of brush borders, as well as flattening of tubular cells. Some tubules demonstrated cell swelling and vacuolization. Kidneys perfused with pRBC depicted the anisometric vacuolization of tubular cells, with more flattened tubules and some single large vacuoles. Interstitial hemorrhages, thrombi or vascular injuries were not observed. Necrosis was only observed in some single spots in Ecosol perfused kidneys. The tubular injury severity score did not differ significantly between the groups before perfusion (0.75 ± 0.5 in pRBC vs. 0.75 ± 0.5 in Ecosol). However, kidneys in the Ecosol group scored significantly higher than kidneys in the RBC group after 6 h perfusion (Figure 8c; *p =* 0.0152) with a mean tubular injury severity score of 2.57 ± 0.45 compared to 2.00 ± 0.00. The extent of tubular vacuolization was comparable between the groups (0.75 ± 0.5 in pRBC vs. 1 ± 0.63 in Ecosol) before perfusion and higher (*p* < 0.0001) in pRBC after perfusion, with a mean score of 4.00 ± 0.00 compared to Ecosol.

## 4. Discussion

Scientific efforts regarding preservation and conditioning techniques for organ transplantation are increasing. Among working groups, a wide spectrum of different and complex perfusion protocols exists, demanding simpler solutions. Considering the opportunity of organ conditioning during NMP, perfusion solutions aiming to ameliorate tissue damage, such as Ecosol, could offer a great benefit in restoring organ viability.

Here, we compared Ecosol, a cell- and oxygen carrier-free preservation solution, containing the potent antioxidant taurine, to the clinically applied pRBC-based buffer solution for 6 h NMP of porcine kidneys. The major and novel finding is that Ecosol was able to provide stable perfusion parameters, such as RBF, pH, and constant urine production during 6 h of NMP. Most importantly, oxygenation was sufficient even without an oxygen carrier, preventing the shift to hypoxic conditions. This was indicated by comparable perfusate pO_2_ levels and HIF-1α levels in both groups. The overall kidney damage in the Ecosol group was, as represented by AST and lactate perfusate levels, comparable to the pRBC group, and better with regard to LDH and urine protein concentrations. The antioxidative potential of Ecosol, provided via taurine, was better than the pRBC-based perfusate, as expressed by a higher antioxidative capacity (AC) and lower oxidation-reduction potential (ORP). The formulation of Ecosol could be improved for NMP, since electrolyte and glucose levels were outside physiological ranges and an insufficient colloid-osmotic pressure was present due to a lower than specified PEG M_w_. This led to extensive tissue edema, the formation of gel deposits on the kidney surface and high IRR, potentially causing aggravation of kidney damage and an increase of apoptosis as shown in WB analysis. Nevertheless, the overall performance of the kidneys after 6 h of NMP was still comparable between the two experimental groups.

NMP has been increasingly employed over the last decades for investigating kidney viability and the restoration of graft function. NMP provides a unique opportunity for functional organ assessment and predicting short-term transplantation outcome [7,8]. However, the variety of different perfusion protocols and lack of established, objectifying parameters impede prediction of long-term transplantation outcomes. Kaths et al. found that perfusate AST might be an important marker of renal graft function, as post-transplantation outcomes correlate with AST levels during NMP [32] and Hosgood et al. implemented a simple scoring system for functional assessment based on macroscopic appearance, arterial blood flow and urine flow [33]. Unifying NMP protocols could facilitate the search for more reliable parameters to assess organ viability. A step towards that goal might be the use of an elementary and ready to use perfusion solution for NMP procedures, such as Ecosol.

Cell free perfusion mediums have been tested previously in various NMP settings and have been proven to be feasible for kidney perfusion [17,18,19]. Minor et al. demonstrated that an Aqix RS-I cell free solution allowed for 2 h end-ischemic machine perfusion, rewarming grafts in a controlled fashion prior to NMP [17]. The Lifor solution was shown to protect rat kidneys from warm ischemia reperfusion injury in situ as well as from cold storage injury [18]. Additionally, beneficial effects of perfusion at room temperature were observed in a porcine DCD kidney model [19]. In line with these findings, an Ecosol perfusate performed comparable to a pRBC-based perfusate for 6 h NMP of DCD kidneys.

The impact of an insufficient colloid-osmotic pressure due to a too low M_w_ of the PEG component in the Ecosol solution used for this study, as demonstrated by FTIR and GPC analysis, was not only expressed by extensive tissue edema and significant weight gain. The soluble creatinine in the perfusate accumulated within the PEG containing gel mass found on the organ grafts, resulting in a lower CrCl compared to the pRBC group. Nevertheless, at 6 h of NMP, the CrCl rates were comparable between the two groups. PEG weights lower than 20 kDa lack the colloidal capacity required for successful NMP and prevention of extravasation of the perfusate. The detrimental effects of M_W_ were demonstrated by Neuzillet et al. [34]. Additionally, the use of higher PEG M_W_ > 10 kDa between 1 g/L to 30 g/L preserves the graft integrity and reduces antigen allorecognition, as shown by Giraud et al. [35]. All these findings are in line with morpholigical changes observed in kidneys with Ecosol in our study.

A main concern of using an oxygen carrier-free solution is the ability to sufficiently provide the kidney graft with oxygen. Due to the use of carbogen over a clinically employed oxygenator, supraphysiological pO_2_ levels were achieved in both pRBC and Ecosol kidneys, resulting in comparable perfusate HIF-1α levels. HIF-1α is a transcription factor regulating oxygen-dependent cell responses. Under hypoxic conditions, the oxygen-sensitive HIF-1α is not ubiquitinated, hence avoiding proteasomal degradation. This leads to accumulation of the transcription factor and binding to specific DNA sequences in hypoxia-regulated genes [36,37,38]. Comparable levels of HIF-1α in Ecosol and pRBC are therefore suggestive of sufficient oxygen supply by the cell- and oxygen carrier-free Ecosol solution. It could be argued that 6 h of NMP may not be long enough for regulating mechanisms to become apparent. However, Rosenberger et al. found that following hypoxic conditions, HIF-1α upregulation becomes evident within approximately 2 h [39], thus in our setting, changes in HIF-1α should have been seen.

The proinflammatory cytokine IL-6 plays an important role in inflammatory processes during reperfusion. It is upregulated in the kidney in response to ischemic injury and correlates with adverse transplantation outcomes [40,41]. However, IL-6 levels need to be interpreted cautiously, as it has both pro- and anti-inflammatory properties [40,41,42]. Recently, De Beule et al. found that the cytokine levels between kidneys perfused with whole blood and those perfused with pRBCs did not differ significantly [43]. As pRBCs are deprived of leukocytes, one would assume that the inflammatory response is milder in comparison to whole blood. As that assumption cannot be confirmed by De Beule et al.’s findings, the question is raised if the cytokine expression measured in NMP studies, is not influenced by different perfusates. Accordingly, we did not observe differences, suggesting that IL-6 expression is rather associated with intrinsically available leukocytes than with those added by blood products. Nevertheless, perfusion with Ecosol did not aggravate inflammatory processes more than perfusion with pRBCs.

Acute kidney injury (AKI), as emerging during NMP, is not only associated with inflammation, but with reactive oxygen species (ROS) formation as well [44]. ROS, generated by nicotinamide adenine dinucleotide 3-phosphate oxidase 4 (Nox4), were shown to be crucial in the generation of contrast-induced AKI in in vivo and in vitro models [45]. Ecosol contains taurine as an antioxidant to reduce ROS formation during NMP. To quantify the effect of taurine on oxidative stress in our NMP setting, AC and ORP were measured, according to Rael et al. and Panighrani et al. [46,47]. In the pRBC group, the AC increased almost linearly over time. This finding is explainable by the constant administration of Cernevit, containing antioxidants such as vitamin C, to the pRBC perfusate. The higher AC and decreased ORP in Ecosol compared to pRBC might be due to taurine, only present in Ecosol.

Taurine is a semi-quantitative beta-amino acid and in taurine transporter knockout mice, its antioxidative, antiapoptotic properties were shown [48]. Taurine also proved to be organoprotective in cardiovascular, nervous, retinal, and renal tissues in preclinical and clinical studies [28,29,49,50,51]. Thus, taurine plays a crucial, cytoprotective role in various conditions, such as reperfusion injury [52]. In models of myocardial ischemia and reperfusion injury after coronary bypass surgery, taurine supplementation prior to the intervention showed beneficial effects on the injury size and heart function [49]. Two main arguments led to supplementing kidney NMP with taurine. (1) The renoprotective properties of taurine are well-established [29,53,54]. (2) The supplementation of a preservation solution with taurine and glutathione (GSH) reduced lipid peroxidation and vascular resistance in liver oxygen persufflation preservation models [26,55]. All the components necessary to synthesize GSH are incorporated in Ecosol: glutamate, cysteine, and glycine. In comparison to taurine, the concentrations of GSH components are much lower, so that the effect strength of GSH is postulated to contribute to a much lesser extent to Ecosol’s antioxidative characteristics.

Contrary to expectations, the antioxidative properties were not able to directly mitigate tissue damage and initiation of apoptosis, as shown by the higher tubular injury score and Casp 3 levels in Ecosol compared to pRBC. Similarly, normothermically perfused kidneys did not derive benefits from the addition of vitamin C to the perfusate, as shown by a preceding study [16]. The tubular vacuolization observed mainly in the pRBC group could be linked to hyperglycemia occurring in the pRBC-based perfusate, thus being a sign of osmotic nephropathy. Inducing renal hypoxia, volume depletion and ultimately ROS formation via osmotic diuresis hyperglycemia directly causes renal injury [56,57]. This could have contributed to the lower AC and higher ORP in the pRBC group.

On the protein level, the addition of taurine to Ecosol was reflected by a slightly favorable antiapoptotic status observed in Ecosol kidneys. Casp 3, pAkt/Akt, and pERK/ERK signaling were analyzed in renal tissue samples collected after 6 h NMP. High Casp 3 levels would have been expected to be reflected in more extensive tissue damage. Opposingly, neither the beneficial antioxidant capacities in the Ecosol group, nor the better histological tubular injury score in the pRBC group could be explained by the high Casp 3 levels in the Ecosol group. Cleaved Casp 3 was not detected in WB analysis, although the antibody is specific for both full-length and cleaved Casp 3. Consequently, upregulation of caspase 9 is likely missing, as it is responsible for Casp 3 cleavage [58]. As we did not find effector fragments in neither of the groups, no statement can be made on the apoptotic status of the analyzed renal tissue. However, a better-preserved cell status in Ecosol perfused kidneys could be assumed, as taurine potentially has a predominant role in this mechanism [59]. This leads to the assumption that Ecosol as an acellular perfusate in its present formulation (including low M_w_ PEG), is not superior in terms of cellular damage, but still not inferior to the pRBC-based perfusion in NMP settings, with the potential to be an efficient NMP solution in a modified version. In good agreement with this, no significant differences in pAkt/Akt and pERK/ERK signaling were observed between the groups.

Our study is limited in several aspects. First, the relatively small number of kidney grafts in the experimental groups (n = 7) as well as the use of porcine kidneys calls for the careful interpretation of results. Additionally, our study only assessed kidney function during NMP, and the kidneys were not transplanted. The performance of Ecosol was impaired by containing PEG with a lower M_w_ than specified by the manufacturer, making only general statements to its feasibility possible. The euthanasia protocol probably influenced the perfusate electrolyte concentrations. Lastly, whether kidney function during NMP with a cell- and oxygen carrier-free perfusion will translate into long-term graft function remains to be explored. Although all findings suggest that the use of Ecosol in our NMP setting leads to comparable perfusion outcomes, the relevance of these findings should further be investigated into extended NMP periods and transplantation of the perfused graft.

## 5. Conclusions

The cell- and oxygen carrier-free Ecosol preservation solution has the potential to become an effective NMP perfusate if the colloid-osmotic pressure can be improved by increasing the M_w_ of PEG.

Further studies involving transplantation models are required to elucidate the influence of perfusates and antioxidants used for NMP in relation to post-transplant outcomes.

Taken together, the findings of this study support the aim to optimize a cell- and oxygen carrier-free perfusion solution, since the general feasibility of oxygen carrier-free NMP was demonstrated.

## Figures and Tables

**Table 1:** Mean perfusate electrolyte and glucose concentrations compared to standard values.

	pRBC ^1^	Ecosol ^1^	Standard Value
K^+^ (mmol/L)	6 ± 0.46	12 ± 0.16	3.6–5.2
Na^+^ (mmol/L)	162 ± 1.4	109 ± 0.66	135–145
Cl^−^ (mmol/L)	131 ± 0.66	50 ± 0.14	98–106
Ca^++^ (mmol/L)	0.87 ± 0.12	0.69 ± 0.03	1.15–1.35
Glucose (mg/dL)	328 ± 19	76 ± 8.6	90–140

^1^ Values are presented as mean ± SEM.

## Data Availability

The datasets generated and/or analyzed during the current study are available from the corresponding author upon reasonable request.
